# Proteomics and Metabolomics in Biomedicine

**DOI:** 10.3390/ijms242316913

**Published:** 2023-11-29

**Authors:** Lucia Santorelli, Marianna Caterino, Michele Costanzo

**Affiliations:** 1 Department of Oncology and Hematology-Oncology, University of Milano, 20122 Milan, Italy; lucia.santorelli@unimi.it; 2 Department of Molecular Medicine and Medical Biotechnology, University of Naples Federico II, 80131 Naples, Italy; marianna.caterino@unina.it; 3 CEINGE–Biotecnologie Avanzate Franco Salvatore, 80145 Naples, Italy

The technological advances of recent years have significantly enhanced medical discoveries. In particular, biomedical research has been largely supported by the omics revolution [1]. During this revolution, each omics science has independently grown to deliver branches of knowledge with precious information at different levels, offering new, intriguing resources to integrate such information across these molecular layers [2,3]. Among them, proteomics and metabolomics allow for the description of the layout of the biochemical processes and organization of the human body, in both pathological and physiological contexts. At present, proteomics and metabolomics approaches for biomedicine rely on the most advanced liquid chromatography–tandem mass spectrometry (LC–MS/MS) platforms despite many metabolomics investigations being more suitably carried out using gas chromatography (GC)–MS or nuclear magnetic resonance (NMR) systems for molecule detection and identification [4,5,6].

Relevant metabolic information can be tracked through investigation of the alterations in the proteome or the sub-proteomes of cells or organisms, with clear appreciation of the variations occurring in signaling pathways or interactome networks, as described for cancer, metabolic disorders, or infective diseases [7,8,9,10,11,12,13,14]. To obtain proteome-wide information on proteins and their post-translational modification status, even at the single-cell resolution, protein quantification strategies have been developed, with them mostly relying on MS properties [15,16,17,18,19,20]. On the other hand, metabolomics technologies have helped the discovery of several potential biomarkers of diseases pertaining to renal, intestinal, or hepatic injury, particularly originating from studies performed on biological fluids—so-called “liquid biopsies”—such as saliva, sweat, breath, semen, feces, and amniotic, cerebrospinal, and broncho-alveolar fluid [21,22,23]. Metabolomics generally focuses on small molecules belonging to the hydrophilic classes, while lipidomics has emerged as an independent omics owing to the complexity of the lipid species [24,25,26].

On the verge of achieving conclusive results in biomedicine with the support of omics sciences, many questions related to the most disparate fields remain unsolved. Accordingly, this topic brings together thirty-eight published articles unveiling interesting findings with potential biomedical and clinical applications.

Metabolomics approaches have been largely adopted to investigate the changes in non-alcoholic fatty liver disease associated with therapeutic responses [27], to identify known and novel circulating metabolites and metabolic pathways associated with coronary heart disease [28], to find alterations in acylcarnitine levels in the brain of mice with neonatal hypoxic–ischemic brain injury [29], to explore the causes of low serum 25-hydroxyvitamin D after living donor liver transplantation [30], and to analyze the fecal lipidomic profiles of individuals with metabolic syndrome [31]. Moreover, with an application to cancer research, Buszewska-Forajta et al. explored the possibility of using citric acid as a potential biomarker for prostate cancer [32].

Other studies have been conducted with a precise focus on pregnancy and embryonic development. In particular, a metabolomic analysis performed on placenta and cord blood showed deep lipid and energetic metabolic remodeling due to intrauterine growth restriction [33]; another study focusing on the differences between the arterial and venous umbilical cord plasma metabolome pointed out a particular association between venous metabolites and parity, clarifying the importance of the choice of mixed, arterial, or venous cord blood when performing experiments related to this field [34]. In animals, cyclophosphamide was found to induce lipid and metabolite perturbation in amniotic fluid during rat embryonic development [35]; in contrast, the effect of sex steroids on pregnancy-associated hormone receptors and extracellular matrix proteins was evaluated during early–mid pregnancy in cows [36].

Non-human organisms have been analyzed by some authors, resulting in the identification of two proteins, glucose/ribitol dehydrogenase and 16.9 kDa class I heat shock protein 1, as novel wheat allergens [37], or the obtainment of insights into triterpene saponin biosynthesis in the tea plant (*Camellia sinensis*) [38]. Moreover, the effects of different molecules have been investigated, such as the antiviral effects of *Isatis* root polysaccharides against porcine reproductive and respiratory syndrome virus [39] or the possible anticoagulant and antithrombotic effects of the venom of the snake *Deinagkistrodon acutus* [40]. Potential pharmacological effects were also found by Xie et al. who associated the antipyretic mechanism to ellagic acid in rats [41,42]. Additionally, a blood deficiency rat model was used to investigate the hematopoietic and antioxidant effects of Maoji Jiu medicinal wine on metabolism [43].

The same nutraceutical approach was employed by Kim et al. in their demonstration of sulforaphane’s protective activity on secondhand smoking-induced pulmonary damage [44] and Jo et al. who elucidated the downstream effects of donepezil treatment on gut microbiota in Aβ-induced cognitive impairment status [45]. The correlation between proteome or metabolome changes and drug perturbation was also explored in some of the selected papers. A proteomic snapshot of progressive molecular signatures was carried out in hippocampal mice tissue after pharmacological inhibition of nitric oxide synthase activity [46], whilst a proteomic analysis highlighted the effects of chitosan hydrogel supplemented with metformin on the neural differentiation of stem cells, with the final aim of employing them in the construction of biomimetic scaffolds for disease treatment [47].

The methodological aspects of the included studies mostly focused on MS, and its developments and applications have also been investigated. For instance, it was demonstrated that the chosen sample preparation process remarkably impacts tissue collection in MALDI imaging approaches [48], and even the sample transport process affects the proteome of clinically relevant samples such as blood extracellular vesicles [49]. Using differential affinity chromatography coupled to mass spectrometry, an antimicrobial peptide was found to interact with common targets in eukaryotes involved in essential pathways [50]; the authors suggest considering this approach to identify drug targets in pathogens and hosts with the aim of eliminating compounds with potential side effects [50]. To improve pharmacokinetic properties, the modification of molecules, such as PEGylation or fusion to PEG mimetic polypeptides, is important. Two PEG mimetic approaches, GlycoTAIL and FlexiTAIL, were applied to increase the hydrodynamic radius of antibody fragments of different sizes and valencies, with them exhibiting a prolonged half-life and increased drug disposition in mice [51]. One paper proposed three diverse techniques to isolate starch grain proteins, demonstrating how different sample preparation processes influence the respective proteomic results [52]. Godbole et al. developed a severity score for respiratory disorders, such as airflow obstruction and emphysema, based on metabolomics analyses [53]. Finally, Terracina et al. evaluated the reliability of an automated urine dipstick analysis method for creatinine and albumin values with respect to quantitative tests. They found that, despite being more rapid and less expensive, dipstick assessment tends to overestimate the albumin/creatinine ratio, resulting in a higher number of false positives [54].

Nonetheless, a total of seven reviews were also published as part of this topic. Some of these revolve around the application of proteomics and metabolomics to biomarkers or pathway discovery for the diagnosis and management of cancer [55,56,57], while others evaluate the impact of metabolomics in the recognition of peritoneal membrane dysfunctions in patients undergoing peritoneal dialysis [58]. Opportunities and challenges have been discussed with regards to pharmacometabolomics for the study of lipid-lowering therapies [59] and for the enrichment and characterization of protein lipidation as a form of post-translational modification [60]. Finally, one article extensively describes the proteomic and metabolomic signatures of COVID-19, emphasizing the latest discoveries regarding the disease and the efforts provided by the omics community, unifying them under the term COVIDomics [61].
